# Antimicrobial Effects of Black Soldier Fly and Yellow Mealworm Fats and Their Impact on Gut Microbiota of Growing Rabbits

**DOI:** 10.3390/ani10081292

**Published:** 2020-07-28

**Authors:** Sihem Dabbou, Ilario Ferrocino, Laura Gasco, Achille Schiavone, Angela Trocino, Gerolamo Xiccato, Ana C. Barroeta, Sandra Maione, Dominga Soglia, Ilaria Biasato, Luca Cocolin, Francesco Gai, Daniele Michele Nucera

**Affiliations:** 1 Center Agriculture Food Environment (C3A), University of Trento, Via E. Mach 1, 38010 San Michele all’Adige, Italy; sihem.dabbou@unitn.it; 2 Research and Innovation Centre, Fondazione Edmund Mach, 38010 San Michele all’Adige, Italy; 3 Department of Agricultural, Forest and Food Sciences, University of Torino, Largo Paolo Braccini 2, 10095 Grugliasco, Italy; ilario.ferrocino@unito.it (I.F.); ilaria.biasato@unito.it (I.B.); lucasimone.cocolin@unito.it (L.C.); daniele.nucera@unito.it (D.M.N.); 4 Department of Veterinary Science, University of Turin, Largo P. Braccini 2, 10095 Grugliasco, Italy; achille.schiavone@unito.it (A.S.); sandra.maione@unito.it (S.M.); dominga.soglia@unito.it (D.S.); 5 Department of Comparative Biomedicine and Food Science, University of Padova, Viale dell’Università 16, Legnaro, 35020 Padova, Italy; angela.trocino@unipd.it; 6 Department of Agronomy, Food, Natural Resources, Animal, and Environment, University of Padova, Viale dell’Università 16, 35020 Legnaro, Padova, Italy; gerolamo.xiccato@unipd.it; 7 Nutrition and Animal Welfare Service, Department of Animal and Food Science, Faculty of Veterinary Medicine, Autonomous University of Barcelona, Bellaterra, 08193 Barcelona, Spain; Ana.Barroeta@uab.cat; 8 Institute of Sciences of Food Production, National Research Council, Largo Paolo Braccini 2, 10095 Grugliasco, Italy; francesco.gai@ispa.cnr.it

**Keywords:** insect fat, *Hermetia illucens*, *Tenebrio molitor*, gut microbiota, antimicrobial effect, rabbit feeding

## Abstract

**Simple Summary:**

The use of insect lipids as an alternative ingredient is an emergent topic in animal nutrition due to their antimicrobial activities. The present study evaluated the in vitro antimicrobial activities of two insect fats (black soldier fly (*Hermetia illucens* (HI) fat and yellow mealworm (*Tenebrio molitor* (TM) fat) and their effect as a total substitute for dietary soybean oil in cecal fermentation and gut microbiota of growing rabbits. The obtained results showed the potential of HI and TM fats as an antibacterial feed ingredient with a positive influence on the rabbit cecal microbiota. HI and TM fats therefore may be a sustainable lipid alternative to soybean oil in rabbit nutrition with possible interesting applications in the feed industry.

**Abstract:**

This study aimed to evaluate the in vitro antimicrobial activities of two types of insect fats extracted from black soldier fly larvae (HI, *Hermetia illucens* L.) and yellow mealworm larvae (TM, *Tenebrio molitor* L.) and their effects as dietary replacement of soybean oil (S) on cecal fermentation pattern, and fecal and cecal microbiota in rabbits. A total of 120 weaned rabbits were randomly allotted to three dietary treatments (40 rabbits/group) —a control diet (C diet) containing 1.5% of S and two experimental diets (HI diet (HID) and TM diet (TMD)), where S was totally substituted by HI or TM fats during the whole trial that lasted 41 days. Regarding the in vitro antimicrobial activities, HI and TM fats did not show any effects on *Salmonella* growth. *Yersinia enterocolitica* showed significantly lower growth when challenged with HI fats than the controls. The insect fat supplementation in rabbit diets increased the contents of the cecal volatile fatty acids when compared to the control group. A metataxonomic approach was adopted to investigate the shift in the microbial composition as a function of the dietary insect fat supplementation. The microbiota did not show a clear separation as a function of the inclusion, even if a specific microbial signature was observed. Indeed, HI and TM fat supplementation enriched the presence of *Akkermansia* that was found to be correlated with NH3-N concentration. An increase in *Ruminococcus*, which can improve the immune response of the host, was also observed. This study confirms the potential of HI and TM fats as antibacterial feed ingredients with a positive influence on the rabbit cecal microbiota, thus supporting the possibility of including HI and TM fats in rabbit diets.

## 1. Introduction

In rabbit production, a high mortality, which can reach 80%, due to gastrointestinal disorders and epizootic enteropathy is the major health concern [1]. The rabbit’s digestive health and physiology, as well as the immune system, are based on its abundant cecal microbiota [2,3]. The intestinal microbial community is abundant, since it contains about 100 to 1000 billion microorganisms per gram of digesta. Its diversity and complexity is very high, with about a thousand different species. Bacteria predominate with a 10^11^ to 10^12^ bacteria/g cecal content [4]. Cecal microbiota of rabbit species is dominated by phylum Firmicutes (90%), while the other phyla, conventionally found in the digestive ecosystems of mammals, are in the minority (10%). Lachnospiraceae and Ruminococcaceae are the dominant families of the cecal ecosystem (40% and 30%) followed by Bacteroidaceae and Rikenellaceae (less than 3%) [4]. The gut microbiota, as well as the factors affecting its composition, are considered important aspects to maintaining digestive health and therefore to enhancing rabbit production [2,5]. Diet is one of the main factors influencing the microbiota and the co-occurrence patterns of the cecal bacterial community [2,5]. Specifically, dietary fat intake can modulate gut microbiota [6,7]. It has been reported that some fatty acids (FA) and in particular medium chain fatty acids (MCFA) act as bacteriostatic (growth inhibiting) or bactericidal (killing) molecules [8,9]. Then, in addition to modulating the bacterial community, the amount and type of dietary fat can have an effect on the immune function both at systemic and intestinal levels [5]. Data regarding the effect of fat type and level in rabbit diet on gut health are still very limited and lead to contradictory results [5,10].

The well-known antimicrobial resistance caused by the misuse of antibiotics drugs in animal production, and the EU ban on their in-feed use (Regulation EC/1831/2003) has led to an increase in the incidence of livestock disease and economic damage. For several years, research has dedicated great efforts to investigating alternatives able to sustain production without causing an increase of antimicrobial resistance and several natural products have been investigated [11,12].

Recently, insects have been receiving considerable attention as novel alternative feed ingredients because of their excellent nutritive properties [13] and their potential effect on animal health [14,15]. It has been shown that insects can modulate the intestinal microbiota with positive effects on poultry growth, health, and resistance against pathogens [15,16,17,18,19]. Lipids are a main component of insects (30%–40% of the dry matter, DM) [20] and, once extracted during the insect larvae processing, they can be used as an interesting feed ingredient in animal farming [14,15,21,22,23,24,25,26]

Black soldier fly (*Hermetia illucens*, HI) and yellow mealworm (*Tenebrio molitor*, TM) fats are characterized by a different fatty acid (FA) profile. The HI fat is rich in saturated FAs (SFAs) and in MCFAs, mainly lauric acid (C12:0; 48% of the total FA profile) [14], which has an antimicrobial effect on a wide range of microbes [27]. On the other hand, the TM fat is considered as a source of n-6 poly-unsaturated FAs (PUFAs), with high linoleic acid content (9% of the total FA profile) [14]. MCFAs are effectively absorbed and metabolized on the gastro-intestinal tract and known for their physiological and antibacterial effects on Gram positive bacteria [28,29,30]. Furthermore, MCFAs can exert beneficial effects on intestinal health and microbial growth inhibition [31], as well as a favorable impact on the digestive health of the growing rabbit [32]. Both the short chain FAs (SCFAs; propionic acid and butyric acid) and the MCFAs (caproic acid and caprylic acid) have a direct in vitro antimicrobial activity against *Salmonella typhimurium* [33]. Matsue et al. [27] demonstrated that lauric acid has a high antimicrobial activity against pathogenic *Bacteroides* and *Clostridium* species, and consequently can modulate intestinal health. Significant in vitro gut antimicrobial effects against D-*Streptococci* and *Lactobacilli* have also been attributed to HI prepupae fat [30]. In a recent study, Sypniewski et al. [15] showed that the addition of HI fat to replace soybean oil (S) in turkey diets significantly reduced the presence of potentially-pathogenic microbes and decreased gastrointestinal-tract (GIT) inflammation by modulating the level of IL-6 and TNF-α.

In the light of what was reported above, the aim of the present study was to evaluate the in vitro antimicrobial activities of HI and TM fats and their effects on cecal traits and gut microbiota of growing rabbits.

## 2. Materials and Methods 

### 2.1. Animal Ethics Statement

The study was performed in accordance with the guidelines of the European and the Italian laws (European Directive 86 609/EEC-Italian law D.L. 116/92), and was approved by the Bioethical Committee of the University of Torino (Italy) (Ref. 386638, 4/12/2017).

### 2.2. In Vitro Analyses for Antimicrobial Activity of Insect Fats

Bacterial strains were selected considering their impact on rabbits and on public health [34,35,36,37]. *Salmonella tiphymurium* (CIP 60.62T), *Salmonella enteritidis* (CRBIP 19.329), *Yersinia enterocolitica* (CIP 101.776), *Pasteurella multocida* (CIP 100.610) and *Listeria monocytogenes* (CIP 82.110T) were all purchased from Institute Pasteur (Paris, France). After overnight incubation following producers’ instructions, strains were mixed with TM and HI fats [14] in order to reach a final concentration of 3 Log CFU/mL, verified by immediate plate streaks. Briefly, the broths for growth were prepared as follows: 200 µL of brain heart infusion broth (BHI, Oxoid, Fisher Scientific, Rodano, Milano, Italy) were added to 250 µL of insect fat and then to 50 µL of fresh bacterial overnight culture previously quantified by measuring the optical density at 600 nm (Prixma UV/VIS 5200, Fulltech Instruments, Rome, Italy) and diluted to 4 Log CFU/ml. Only for *Pasteurella multocida*, instead of BHI, tryptic soy broth (TSB, Oxoid, Fisher Scientific, Rodano, Milano, Italy) was used. These mixes were incubated 24 hours at 37 °C with horizontal shaking (RPR horizontal rotator, LE8S, Fisher Scientific, Rodano, Milano, Italy). At regular timings (every 2 hours), three aliquots for each mix were streaked on tryptic soy agar (TSA, Oxoid, Fisher Scientific, Rodano, Milano, Italy) (only *Pasteurella multocida*) or on brain heart infusion agar (BHA, Oxoid, Fisher Scientific, Rodano, Milano, Italy). Moreover, control tests were prepared—450 µL of BHI were added with 50 µL of quantified overnight cultures to reach a final concentration of 3 Log CFU/mL of broth (A) and a mix of soybean oil (S) and bacterial broth, prepared as described above for insect fats (B). Each control and strain/fat combination were analyzed in triplicates for a total of 84 samples per bacterial species—12 samples per 6-time intervals. 

### 2.3. Inclusion of Hermetia illucens and Tenebrio molitor Fats in the Diet of Growing Rabbits

#### 2.3.1. Experimental Design

For this experiment, three dietary treatments were tested in 120 growing rabbits (40 rabbits/diet) from 36 to 78 days of age—a control diet (C) containing 1.5% soybean oil (S), and two diets (HID and TMD) with total replacement of S with HI and TM larvae fat, respectively. TM and HI fats were obtained from commercial companies (Ynsect, Evry, France and Hermetia Deutschland GmbH & Co. KG, Baruth/Mark, Germany) by a mechanical process using high pressure and without solvents. The three diets contained an average of 16.6% DM of crude protein (CP) and 18.6 MJ/kg DM of gross energy. Detailed information about the rabbit farming conditions and live performance are provided by Gasco et al. [14]. Ingredients and chemical composition of the diets are shown in Table 1. Briefly, no overall significant differences among experimental groups were observed for growth performance during the trial. 

#### 2.3.2. Fatty Acid Profiles of Insect Lipids and Experimental Diets

The FA profiles of insect lipids and feeds were determined as reported by Gasco et al. [14]. The fatty acid methyl esters (FAME) were separated, identified, and quantified. The results were expressed as g/kg of FAME (Table 2).

#### 2.3.3. Cecal Fermentation Traits

At slaughtering (78 days of age), a total of 30 rabbits (10 animals per treatment) were randomly selected and eviscerated. The pH, cecal ammonia, and volatile FA (VFA) profiles of the cecal contents were determined as reported by Tazzoli et al. [38].

#### 2.3.4. DNA Extraction and 16S rRNA High-Throughput Amplicon Target Sequencing

In order to observe the development and dynamics of bacterial communities, hard feces were collected from 10 rabbits per group at 36, 51, and 77 days of age, whereas the cecal contents (n = 10 per group) were taken during the slaughtering at 78 days of age. Samples from the same group and the same collection site and day were pooled together in sterilized polyethylene bags, and immediately stored at −80 °C until examination. DNA from feces and cecal samples were extracted by using a commercial kit (QIAamp Fast DNA stool Mini Kit, QIAGEN®, Hilden, Germany) following the instructions reported by the manufacturer with slightly modification. The DNA were quantified by NanoDropTM 2000 Spectrophotometer (Fisher Scientific, Rodano, Milano, Italy) and standardized at 5 ng/uL and used a template in the PCR amplifying the V3–V4 region of the 16S rRNA gene using the primers and protocols described by Klindworth et al. [39]. The PCR amplicons were cleaned according to Illumina (San Diego, CA, USA) guidelines. The sequencing was performed with a MiSeq Illumina instrument with V3 chemistry and generated 250 bp paired-end reads according to the manufacturer’s instructions.

#### 2.3.5. Bioinformatics and Statistical Analysis

After paired-end sequencing, raw reads were analyzed as previously reported by Biasato et al. [17]. Sequences were first joined using FLASH software [40] with default parameters and filtered for low quality bases (at Phred < Q20) by QIIME 1.9.0 software [41]. Reads shorter than 300 bp were discarded by using Prinseq software [42]. The USEARCH software version 8.1 was used for chimera filtering and operational taxonomic units (OTUs) were clustered at 97% of similarity threshold by UCLUST algorithms [43]. Representative sequences of each cluster were mapped against the Greengenes 16S rRNA gene database version 2013 for taxonomic assignment. In order to avoid bias due to the different sequencing depth, OTU tables were rarefied at 3996 sequences/sample. tables display the higher taxonomy resolution that was reached. When the taxonomy assignment was not able to reach the genus level, the family or phyla were displayed. Alpha diversity indices were calculated using the diversity function of the vegan package [44]. Weighted and unweight UniFrac distance matrix and OTU tables were used to find differences between samples by anosim and adonis statistical tests through the function vegan in R environment. The pairwise Wilcoxon test was used as appropriate to determine significant differences in alpha diversity or OTU abundance. A generalized linear model was used in order to test the importance of continuous or discrete variables available (sampling time and insect inclusion) on the relative abundance of bacterial genera or family. The interactions between the levels of the fixed factors were evaluated by pairwise comparisons. Not-normally distributed variables were presented as median (range interquartile) and box plots represented the interquartile range between the first and the third quartiles, with the error bars showing the lowest and the highest value. Pairwise Spearman’s non-parametric correlations were used to study the relationships between the relative abundance of microbial taxa in cecal samples and VFA variables. The correlation plots were visualized in R using the coplot package of R. 

The statistical analysis for data related to in vitro antimicrobial activity and cecal traits was performed using IBM SPSS Statistics V25.0.0 software (IBM, Armonk, NY, USA) by means of ANOVA, followed, if significant, by Duncan test post-hoc. Regarding the antibacterial activities of insect fats, bacterial counts was evaluated at each time point and the average results compared across the different conditions—pure bacterial broth, bacterial broth mixed with soybean oil, bacterial broth mixed with TM fat, and bacterial broth mixed with HI fat. Significance was declared at *p* ≤ 0.05. A statistical trend was considered for *p* ≤ 0.10. Sequences data were deposited on NCBI database under the bioproject number PRJNA645756.

## 3. Results

### 3.1. In Vitro Antimicrobial Activities of Insect Fats

Overall, the bacterial counts, when quantified broths were challenged with insect fats, were lower than the controls for three out of five pathogenic species tested, whereas only the two strains of *Salmonella* did not show any significant difference in counts when compared to control tubes counts (therefore these data are not reported in Table 3). Considering the other species, for all of them results highlighted that HI fat caused a delay in bacterial growth, implying a bacteriostatic effect (Table 3). After 24 hours of incubation, counts of *Pasteurella moultocida* broths challenged with HI fat showed a mean log difference of −4.48 and −4.76 when compared to control broths with soybean oil (control B) and no oil addition (control A), respectively. Similar results were observed for *Yersinia enterocolitica* showing a mean log difference values of −5.9 and −5.97, with control B and A, respectively. Finally, *Listeria monocytogenes* counts also led to a similar pattern of results, showing mean log difference values of −5.11 and −5.15 when comparing counts of broths challenged with HI fat and the controls B and A. All these differences were statistically significant (Table 3). On the other hand, results related to TM fat showed that only *Pasteurella multocida* was effectively inhibited in growth—the mean log difference between controls and TM-challenged broths showed values of −2.64 and −2.92, respectively, for control B and control A. These values were lower than what reported above for broth challenged with HI fat (Table 3). Finally, differences in bacterial counts between HI fat challenged broths and controls were statistically meaningful from the 4th hour of incubation only in the case of *Yersinia enterocolitica*, whereas for the other species the bacteriostatic effects were achieved only from the 8th hour of incubation. TM-fat-challenged broths showed significant effect only in *Pasteurella mutocida* after 8 hours of incubation and this was maintained until the end of the experiment (see details in Table 3).

### 3.2. Cecal Fermentation Traits

The cecal fermentation traits are reported in Table 4. The supplementation of insect fat increased the total VFA contents when compared to the control group (85.3 vs. 83.9 vs. 72.4 mmol/L; *p* < 0.05), whereas the pH, the cecal ammonia-N content, and the molar proportion of the different VFAs and their ratio were not influenced by the supplementation of insect fats (*p* > 0.05).

### 3.3. Cecal and Fecal Microbiota Characterization

**Figure 1:**
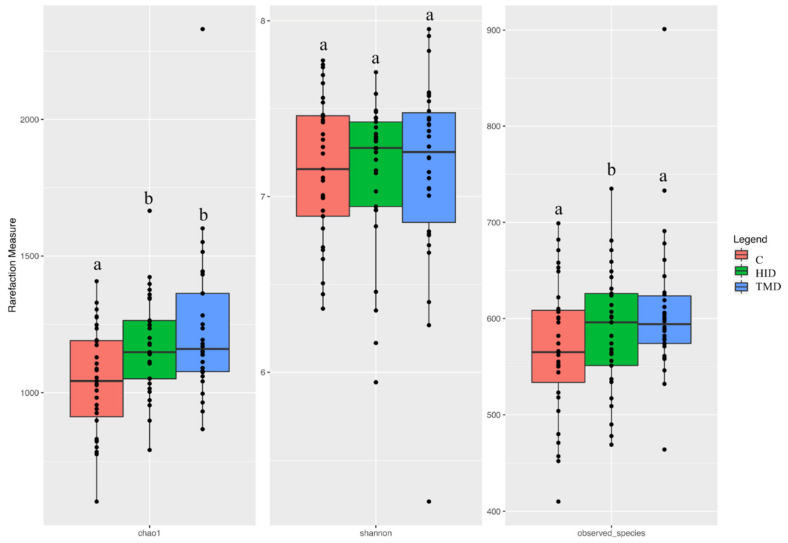
Boxplots showing the alpha diversity rarefaction index across fecal samples of rabbits fed with TMD (blue box), HID (green box), or C (red box) diets; C: control diet with soybean oil; HID: diet with *Hermetia illucens* fat; TMD: diet with *Tenebrio molitor* fat; Boxes represent the interquartile range (IQR) between the first and third quartile and the line inside represents the median (2nd quartile). Whiskers denote the lowest and the highest values within 1.56 IQR from the first and third quartile, respectively. Different letters in each box indicate significant difference.

The total number of high-quality paired-end sequences obtained from 16S rRNA sequencing reached 13,448,661 raw reads. After the filtering, 7,801,336 reads passed the filters applied through QIIME, with a median value of 59,114 ± 52,946 reads/sample, and a mean sequence length of 441 bp. The rarefaction analysis and Good’s coverage, expressed as a median percentage (93%), also indicated satisfactory coverage for all the samples. We applied a generalized linear model (GLM) for the alpha-diversity in the fecal samples in order to test the effect of the treatment across time. The Chao1 index increased (*p* < 0.01) in the TM and HI groups when compared to the C group (*p* < 0.05), while the Shannon index was affected by the sampling time only. However, the number of observed species significantly increased in the HI group in comparison with the other diets (*p* < 0.05) (Figure 1).

No significant differences were observed when comparing the alpha diversity index as a function of the different diets in the cecal samples. Going more deeply in the microbiota comparison, adonis and analysis of similarity (ANOSIM) statistical tests, based on the weighted and unweighted UniFrac distance matrix showed significant differences among samples as a function of the sampling time (*p* < 0.05). However, the dietary inclusion of insect fat did not show any significant effect in the microbiota composition of the cecal samples. 

**Figure 2:**
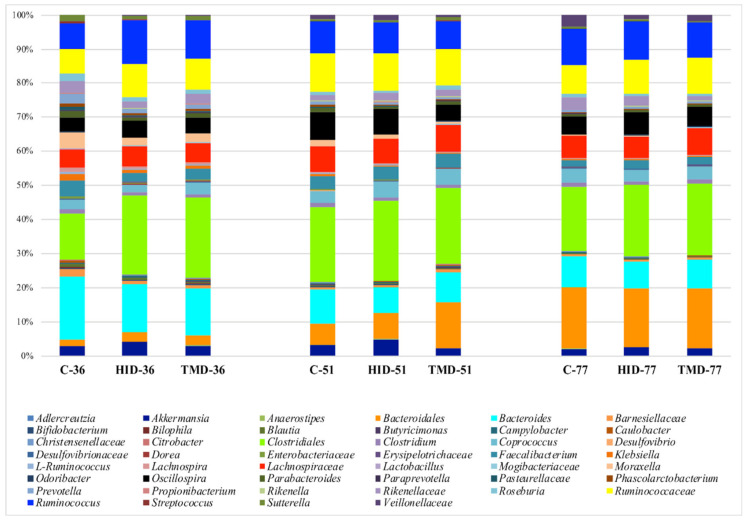
Taxonomic groups detected in fecal samples by means of 16S amplicon target sequencing. Only operational taxonomic units (OTUs) with an incidence above 0.2% in at least two samples are shown. The samples are labelled according to the type of fat supplementation (C: control diet with soybean oil; HID: diet with *Hermetia illucens* fat; TMD: diet with *Tenebrio molitor* fat) and sampling time (36, 51, or 77 days).

Figure 2 summarizes the distribution of the microbiota across time and samples and displays a simple microbiota composition dominated by the presence of *Bacteroides*, Clostridiales, Lachnospiraceae, Ruminococcaceae and *Ruminococcus*. Comparing the relative abundance of the main OTUs across the fecal samples grouped as a function of the dietary supplementation by the GLM function, it was possible to observe that HI inclusion level increased the relative abundance of *Akkermansia* (*p* < 0.05) and *Ruminococcus* (*p* < 0.01) and reduced the presence of *Citrobacter* (*p* < 0.05).

**Figure 3:**
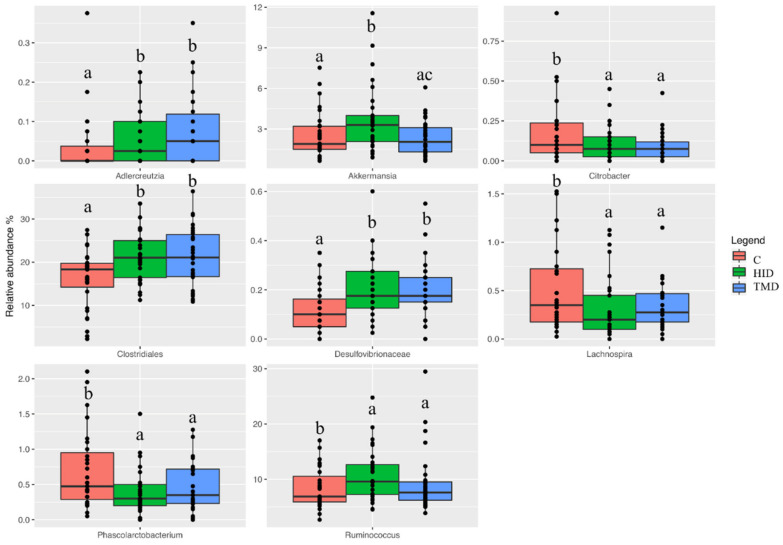
Boxplots showing the relative abundance at genus or family level of operational taxonomic units (OTUs) differential abundant based on the generalized linear model (GLM) test in fecal samples of rabbits fed with TMD (blue box), HID (green box), or C diets (red box). Different letters in each box indicate significant difference; C: control diet with soybean oil; HID: diet with *Hermetia illucens* fat; TMD: diet with *Tenebrio molitor* fat.

Both HID and TMD increased the relative abundance of Clostridiales (*p* < 0.01) and Desulfovibrionaceae (*p* < 0.01), while reducing the relative abundance of *Lachnospira* (*p* < 0.05) and *Phascolarctobacterium* (*p* < 0.01) when compared to the C diet (Figure 3). 

**Figure 4:**
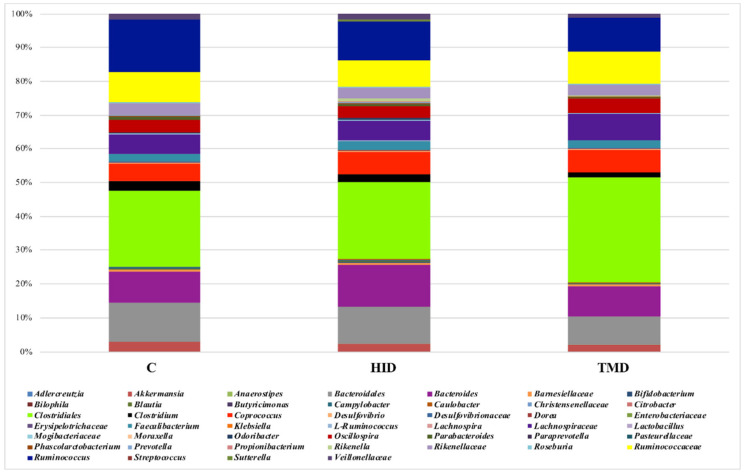
Taxonomic groups detected in cecal samples by means of 16S amplicon target sequencing. Only operational taxonomic units (OTUs) with an incidence above 0.2% in at least two samples are shown. The samples are labelled according to the type of fat supplementation, i.e., TMD, HID, or C diets; C: control diet with soybean oil; HID: diet with *Hermetia illucens* fat; TMD: diet with *Tenebrio molitor* fat.

**Figure 5:**
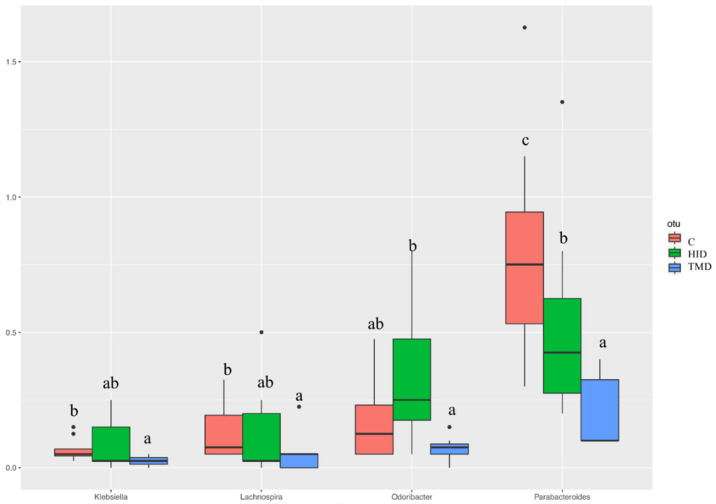
Boxplots showing the relative abundance at genus or family level of the operational taxonomic units (OTUs) differential abundance based on Wilcoxon rank sum test in cecal samples of rabbits fed with TM (blue box), HI (green box), or C (red box) diets. Different letters in each box indicate significant difference; C: control diet with soybean oil; HID: diet with *Hermetia illucens* fat; TMD: diet with *Tenebrio molitor* fat.

Taking into account the cecal samples (Figure 4), no differences were observed regarding the microbial composition and distribution. However, the pairwise comparisons using the Wilcoxon rank sum test showed that TM inclusion reduced the relative abundance of *Klebsiella*, *Lachnospira, Parabacteroides,* and *Odoribacter* compared to the C and HID (Figure 5, *p* < 0.05).

**Figure 6:**
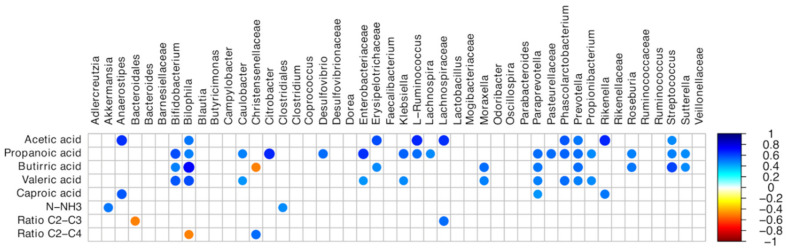
Spearman’s rank correlation matrix of cecal operational taxonomic unit (OTU) abundance and the volatile fatty acid profile. The colors of the scale bar denote the nature of the correlation, with 1 indicating a positive correlation (blue) and -1 indicating a negative correlation (dark red), between the two datasets; N–NH3: ammonia-N; C2: Acetic acid; C3: Propanoic acid; C4: Butyric acid.

The correlations between VFA contents and microbiota are summarized in Figure 6. In detail, strong positive correlations between *Lachnospira* and propanoic acid, *Akkermansia*, Clostridiales, and NH3-N, and *Phascolarctobacterium* and acetic, propanoic, and caproic acids were detected (*p* < 0.05). 

## 4. Discussion

The current study was conducted to evaluate the in vitro antimicrobial properties of HI and TM fats and their effect as an alternative lipid sources in rabbit diets. The possible utilization of insect fats in animal diets has been investigated recently, and so far, a few papers focusing on antimicrobial activities in vitro or in vivo are available, but no data are present in the literature on pathogen growth rate in presence of insect fats. Mustafa et al. [45] showed that the oil extracted from the melon bug (*Aspongopus vidulatus*) was able to inhibit the growth of bacterial species by using the agar well diffusion method, with only Gram positive bacteria (*Staphylococcus, Bacillus,* and *Enterococcus*) being susceptible to crude oil extracts. *Salmonella paratyphi* was also tested and no inhibition was recorded, similarly to what was observed in the present research with other serovars. A recent study of Spranghers et al. [30] on the in vitro effects of fats extracted from HI (from prepupae fed to weaned piglets) pointed out that D-*Streptococci* and *Lactobacilli* were the only bacterial populations that reduced their load after being challenged with insect fats, whereas no effects were recorded on Gram negative bacteria. The data presented in our paper suggest that fats extracted from TM and HI are able to delay bacterial growth of both Gram positive and Gram negative pathogenic bacteria, even if the susceptibility changes with the considered species. Interestingly, no bactericidal effect was observed, thus indicating the possibility of bacterial cells repairing damage that may be induced from FAs or monoglycerides [8]. The antimicrobial effect of TM fat was lower than that of HI fat. These results may be related to the different concentrations of SFA. Indeed, the HI fat used in this study was composed by 79% of SFA (25% in TM fat), and the major component was lauric acid, already reported as very effective against many bacterial species [8,30]. In a systematic study of the in vitro antimicrobial activities of FAs, monoglycerides, and diglycerides, Kabara et al. [46] showed that lauric acid was the most active FA against gram-positive bacteria. On the other hand, the antibacterial effects of unsaturated fatty acids (UFA) reported in literature [8] were not detected in this study, probably in relation to the prolonged incubation time and the temperature of 37 °C, which may have been responsible for the reduction/impairment of the activity of these molecules that characterize TM fat (75% of the FA vs. 21% of HI fat). This hypothesis may also explain why soybean oil did not show any activity, considering its higher level in monounsaturated fatty acids (MUFAs) and PUFAs among all fats used in this work. 

The present observations highlighted the possibility of using insect fats in feed formulation, considering that they may be important for controlling growth of important microbial pathogens such as *Listeria monocytogenes, Yersinia enterocolitica,* and *Pasteurella multocida* that may be important pathogens for rabbits [34,37] or part of the gut flora potentially contaminating rabbit meat during slaughtering [35,36]. Reducing microbial loads in the rabbit gut, apart from animal welfare and safety implications, may also be important for food safety management during slaughtering operations. Studies performed on piglets fed with diets rich in FAs showed a decreased load of microbes in the stomach and gut, mostly if undesired species were considered (i.e., *streptococci*), thus promoting gut heath and growth performances [30,47]. However, as already emphasized by Spranghers et al. [30], more studies need to be performed in vivo, in order to assess the activity of fat in the gut, considering that the bacterial activities and the digestive enzymatic systems of the rabbit (i.e., lipases) may neutralize the FAs, therefore limiting their activity. Furthermore, the presence of FAs as antimicrobial molecules in feed may prevent the use of classical antibiotics in meat production, limiting the diffusion of microbes harboring antimicrobial resistance genes. In fact, antimicrobial resistance mechanisms were rarely observed in microbial species challenged with FAs [47], and to date no genetic modifications induced by exposing bacterial cells to antimicrobial FAs have been shown [8,48].

As far as the in vivo trial is concerned, there was a lack of differences among groups in cecal fermentation traits in our study. Cecal pH and VFA content are the main variables characterizing the extent and the pattern of cecal fermentation, thus constituting an indirect estimate and an important tool to qualitatively evaluate the cecal microbial activity. The dietary HI or TM fat inclusion led to a greater total VFA content in the cecum than that of control diet, thus potentially enhancing the gut with a modification of the fermentation patterns and the composition of the cecal microflora. Peeters et al. [49] previously observed that a high concentration of total cecal VFA in rabbits had a protective effect against enteropathogenic *Escherichia coli* infection. However, the molar proportion of different VFA was not affected by the total replacement of soybean oil with HI and TM larvae fats. 

The present study is the first to investigate gut microbiota of growing rabbits fed diets supplemented with insect fats. As reported previously, HI fat was rich in MCFAs which have a higher antimicrobial activity and thus can stimulate gastrointestinal tract health through inhibition of potentially-pathogenic bacteria [31,33]. MCFAs, and in particular lauric acid, are presumably absorbed in the upper digestive tract, and might be useful in protecting against microbial infection, modulating inflammation, healing wounds, and controlling the balance and distribution of bacteria in gut microbiota [50]. The present study is the first to investigate gut microbiota of growing rabbits fed diets supplemented with insect fats. The results of this study revealed an enrichment of different taxa according to the dietary treatment and a similar microbial diversity and richness between feces and cecum samples. Cecal microbiota is a primary determinant for rabbit health, whereas the fecal microbiota provides an accurate method for studying the evolution of rabbit gut microbiota from weaning to slaughtering [51,52]. Investigating the differences between rabbits fed the C and the HI and TM fat diets, in the current study, no differences were found with regard to alpha-diversity measures in cecal and fecal samples. However, we observed a higher microbial diversity in the HI and TM groups. High levels of diversity generally help intestinal microbiota to determine effective colonization resistance against potential invading pathogens and to modulate animal reaction after a stress-environment [53]. Based on such considerations, the above-mentioned findings are indicative of a positive HI and TM fat-related effect on the gut microbiota of rabbits. *Firmicutes, Bacteroidetes,* and *Verrucomicrobia* represented the dominant bacterial phyla in the control and insect-fat-fed rabbits of the present study. These findings overall agree with previous research that identified *Firmicutes* and *Bacteroides* as the main bacterial phyla in the gut microbiota of rabbits [3,52,54,55]. In relation to the genera composition, the *Bacteroides*, Bacteroidales, Clostridiales, Lachnospiraceae, Ruminococcaceae, and *Ruminococcus*, mainly colonized the cecal and fecal microbiota of the rabbits fed soybean oil or insect fats in the current study. These findings are also in agreement with previous studies, which observed *Bacteroides* [52,55], Clostridiales [54], and *Ruminococcus* [53,56] as being the main bacterial genera in cecal and fecal microbiota of rabbits and being capable of degrading the polysaccharide and amino acid fermentation to produce VFAs. These findings could be the reason for its overrepresentation in cecum where it is supposed to play an active role [52,57]. A previous study reported that the presence of this taxa was positively correlated with the feed conversion rate [58].

Regarding the microbial composition, we did not observe any strong effects as a consequence of the dietary inclusion of HI and TM fats. However, a signature in the microbial population was observed. The fat of TM reduced some taxa such as *Klebsiella*, *Lachnospira, Parabacteroides,* and *Odoribacter*. On the other hand, the dietary supplementation of HI and TM fats enriched the presence of Clostridiales, Desulfovibrionaceae, *Ruminococcus,* and *Akkermansia*, which are the main taxa in the gut microbiota of rabbits [51]. It is well reported that *Akkermansia* can be considered a new-generation probiotic, able to degrade the mucin in the gut with the production of beneficial molecules like SCFAs thus exerting a significant improvement in the gut barrier and in the maintenance of intestinal health [59,60,61]. In addition, it was suggested that *Akkermansia* have an important role in the hydrolysis of various dietary polysaccharides, contributing to increase cellulose digestibility as well as methane metabolism [62,63,64]. *Akkermansia* would be also involved in carbohydrate digestion and in immune protection against inflammation [54]. The increase of this taxon related with the insect fat inclusion suggests an optimal gut environment in our rabbits, even if this observation needs further investigation to be confirmed. In addition, a strong positive correlation between *Akkermansia* and NH3-N was observed. This result confirmed that gut microbiota is responsible for a variety of metabolic activities including production of biologically-active substances. The Ruminococcaceae family is considered as an important producer of short-chain FAs (mainly butyrate, acetic, and succinic acids) through glucose metabolism and cellulose digestion [65,66]. It was reported that members of Ruminococcaceae are important components of the beneficial microbiota of several herbivores [67,68]. Their presence is related to an improvement of the immune system of the host via intestinal mucus degradation and a prevention of acidosis via lactate degradation [69]. Ruminococcaceae has been associated with antibiotic biosynthesis in a metatranscriptomic study on the human gut microbiota, suggesting a role in gut health [70]. The supplementation of HI fat also increased the presence of *Ruminococcus* (belonging to Lachnospiraceae family), that are butyrate-producing bacteria. This ability was also confirmed by the strong positive correlation between L-*Ruminococcus*, acetic, and propionic acid. The identification of short-chain FA-producing bacteria allows us to hypothesize a similar way of action for HI fat in the rabbit’s gut. Therefore, the increase in the above-mentioned bacterial taxa by dietary HI fat may have helped the rabbits to maintain a healthy gut. The microbial signature driven by the inclusion of insect fat increases the presence of *Bacteroides*, *Clostridium, Akkermansia,* and *Ruminococcus* and suggests that dietary HI and TM fats may exert a positive influence on the cecal microbiota of rabbits. It should be pointed out that rabbits fed HI and TM fats showed no significant alterations at histopathological level and no differences in growth performance [14]. However, since a clear cause–effect relationship between diversity and composition of cecal microbiota and rabbit performance has not yet been confirmed, the gut microbiota findings need to be contextualized to underline the effect of insect fats in rabbit diets.

## 5. Conclusions

In conclusion, the results of the present study provide new information about the in vitro antibacterial properties and the in vivo effects at the gut level of the replacement of soybean oil with HI and TM larvae fat in rabbit diets. The in vitro activities of HI or TM fats against *Pasteurella, Yersinia*, and known pathogens of the rabbit gut, indicate a potential for impairing their growth in vivo in rabbits. Furthermore, the dietary inclusion of HI and TM fats stimulate VFA production at the cecum and may positively modulate the cecal and fecal microbiota of growing rabbits. However, further research is needed to confirm the antimicrobial potentiality of insect fats in rabbit feeding.

## Figures and Tables

**Table 1:** Ingredients (% as fed) and chemical composition (% DM) of experimental diets. (modified from Gasco et al. [14]).

	Experimental Diets		
Ingredients	C	HID	TMD
Dehydrated alfalfa meal (17 g CP/100 g)	32	32	32
Alfalfa hay	7.5	7.5	7.5
Wheat bran	23.5	23.5	23.5
Barley meal	10	10	10
Dried sugar beet pulp	16	16	16
Soybean meal (44 g CP/100 g)	7	7	7
Soybean oil	1.5	-	-
*Hermetia illucens* fat	-	1.5	-
*Tenebrio molitor* fat	-	-	1.5
Cane molasses	1.2	1.2	1.2
Dicalcium phosphate	0.3	0.3	0.3
Salt	0.4	0.4	0.4
L–methionine (98 g methionine/100 g)	0.1	0.1	0.1
Vitamin–mineral premix^a^	0.5	0.5	0.5
**Chemical Composition**			
Dry matter, %	89.4	89.2	89.6
Ash, % DM	8.58	7.77	7.75
Crude protein, % DM	17.0	16.8	16.3
Ether extract, % DM	4.22	3.92	3.87
Neutral detergent fiber (aNDF), % DM	40.2	41.7	40.5
Acid detergent fiber (ADF), % DM	21.7	23.0	22.8
Acid detergent lignin (ADL), % DM	4.81	5.09	5.02
Gross energy, MJ/kg DM	18.50	18.50	18.62

C: control diet with soybean oil; HID: diet with *Hermetia illucens* fat; TMD: diet with *Tenebrio molitor* fat; CP: crude protein; DM: dry matter. ^a^Supplementation per kilogram of feed: vitamin A, 16,000 IU; vitamin D3, 1600 IU; vitamin E acetate, 30 mg; vitamin B1, 0.8 mg; vitamin B6, 1.65 mg; niacin, 40 mg; folic acid, 1 mg; Mn, 30 mg; Fe, 116 mg; Cu, 12.5 mg; Zn, 60 mg; Co, 0.45 mg; Ca, 1.3 mg; Se, 0.3 mg.

**Table 2:** Fatty acid profiles of the dietary fats and experimental diets (g kg-1 of total FAME) (modified from Gasco et al. [14]).

Fatty acids	Dietary Fats			Experimental Diets		
	S	HI	TM	C	HID	TMD
C12:0	0.2	480	2.3	0.5	203	3.0
C14:0	0.5	103	22.2	0.9	44.7	13.3
C16:0	104	127	176	121	161	184
C18:0	44.3	19.0	23.1	28.4	20.8	22.2
BCFAs	0.1	2.9	0.8	2.4	4.1	3.4
C16:1 n-7	0.9	32.0	16.6	0.12	19.9	10.4
C18:1 n-9	230	91.1	378	201	127	273
C18:2 n-6	515	90.0	332	521	310	389
C18:3 n-3	70.3	10.1	18.0	74.3	62.8	55.1
SFA^1^	158	748	231	165	454	240
UFA^1^	842	252	769	835	546	760
MUFA^1^	254	141	411	236	169	309
PUFA^1^	588	111	358	599	377	451
∑n3	70.5	11.7	18.3	74.7	62.8	55.1
∑n6	516	91.1	333	523	311	391

S: soybean oil; HI: *Hermetia illucens* fat; TM: *Tenebrio molitor* fat; C: control diet with soybean oil; HID: diet with *Hermetia illucens* fat; TMD: diet with *Tenebrio molitor* fat; FAME: fatty acid methyl ester; BCFAs: branched-chain fatty acids; SFA: saturated fatty acid; UFA: unsaturated fatty acid; MUFA: monounsaturated fatty acid; PUFA: polyunsaturated fatty acid. *^1^*included minor FAs.

**Table 3:** Distribution of average cell counts (Log (CFU)/per mL) over time for the three bacterial species for which inhibition was recorded (means ± SEM; n = 3).

Growth Conditions	*Pasteurella multocida*					
	T4	T6	T8	T10	T12	T24
Control (A)	4.09 (0.19)	4.14 (0.06)	4.76 (0.32)—A	4.80 (0.34)—A	5.69 (0.49)—A	6.82 (0.44)—A
Control (B)	4.11 (0.17)	3.96 (0.12)	4.17 (0.26)—A	4.60 (0.40)—A	5.44 (0.57)—A	6.54 (0.39)—A
TSB + TM fat	3.95 (0.44)	3.75 (0.39)	3.08 (0.35)—B	3.21 (0.30)—B	2.91 (0.86)—B	3.90 (1.21)—B
TSB + HI fat	3.92 (0.20)	3.48 (0.17)	3.61 (0.19)—B	2.80 (0.59)—B	2.67 (0.49)—B	2.06 (0.36)—C
ANOVA	N.S	N.S	F = 4.99; *p* = 0.01	F = 5.07; *p* = 0.01	F = 6.36; *p* < 0.01	F = 13.75; *p* < 0.01
***Yersinia enterocolitica***						
Control (A)	4.36 (0.16)—A	5.19 (0.28)—A	6. 76 (0.61)—A	7.32 (0.37)—A	8.18 (0.37)—A	9.95 (0.30)—A
Control (B)	4.10 (0.21)—A	5.05 (0.37)—A	5.91 (0.41)—A	6.83 (0.48)—A	7.35 (0.68)—A	9.88 (0.33)—A
BHI + TM fat	4.05 (0.31)—A	4.39 (0.52)—AB	4.98 (0.74)—AB	5.04 (0.94)—AB	5.93 (1.41)—AB	8.09 (2.13)—A
BHI + HI fat	3.34 (0.10)—B	2.69 (0.64)—B	3.01 (1.20)—B	3.02 (1.32)—B	2.94 (1.24)—B	3.98 (2.18)—B
ANOVA	F = 3.28; *p* = 0.05*	F = 6.38; *p* < 0.01	F = 4.95; *p* = 0.01	F = 7.71; *p* < 0.01	F = 6.46; *p* < 0.01	F = 5.42; *p* < 0.01
***Listeria monocytogenes***						
Control (A)	4.58 (0.35)	5.58 (0.39)	6.47 (0.41)—A	7.41 (0.41)—A	8.28 (0.46)—A	10.00 (0.26)—A
Control (B)	4.52 (0.32)	5.45 (0.42)	6.26 (0.54)—A	6.94 (0.61)—A	7.95 (0.59)—A	9.96 (0.28)—A
BHI + TM fat	4.20 (0.25)	4.59 (0.79)	5.98 (0.46)—A	6.76 (0.70)—A	7.63 (0.61)—A	9.48 (0.03)—A
BHI + HI fat	4.00 (0.92)	3.66 (0.84)	4.1 (0.63)—B	4.48 (0.61)—B	4.51 (0.62)—B	4.85 (0.97)—B
ANOVA	N.S	N.S	F = 3.30; *p* = 0.05**	F = 3.98; *p* = 0.03	F = 6.85; *p* < 0.01	F = 29.92; *p* < 0.01

HI: *Hermetia illucens*; TM: *Tenebrio molitor*; T: incubation time in hours; TSB: tryptic soy broth; BHI: brain heart infusion; N.S.: non-significant; SEM: standard error of the means. *Significant results (without approximation *p* = 0.046); **results in trend of significance (*p* = 0.051). In the same columns, different letters identify significantly-different results at post-hoc test (Duncan). Control (A) indicates the broth culture without oil/fat in it and control (B) indicates a broth culture grown in BHI/tryptic soy agar and soybean oil.

**Table 4:** Effect of lipid sources on cecal traits and fermentation parameters (n = 10 rabbits/group).

	C	HID	TMD	SEM	*p*-Value
pH	6.1	5.9	5.9	0.01	0.15
N–NH3 (mmol/L)	2.2	3.0	3.1	0.23	0.25
Total VFA (mmol/L)	72.4b	85.3a	83.9a	2.31	0.03
Acetic acid (C2; mmol/100 mmol VFA)	77.8	78.1	76.6	0.43	0.30
Propionic acid (C3; mmol/100 mmol VFA)	5.3	5.0	5.4	0.19	0.71
Butyric acid (C4; mmol/100 mmol VFA)	16.2	16.1	17.2	0.42	0.53
Valeric acid (C5; mmol/100 mmol VFA)	0.5	0.4	0.5	0.03	0.59
Caproic acid (C6; mmol/100 mmol VFA)	0.3	0.3	0.3	0.03	0.45
C2/C3 ratio	15.2	16.0	14.6	0.54	0.58
C2/C4 ratio	4.9	5.0	4.6	0.16	0.63

C: control diet with soybean oil; HID: diet with *Hermetia illucens* fat; TMD: diet with *Tenebrio molitor* fat; SEM: standard error of the means; N–NH3: ammonia-N; VFA: volatile fatty acid.
